# Rheological Properties and Growth Factors Content of Platelet-Rich Plasma: Relevance in Veterinary Biomedical Treatments

**DOI:** 10.3390/biomedicines8100429

**Published:** 2020-10-18

**Authors:** Diego Romano Perinelli, Giulia Bonacucina, Stefania Pucciarelli, Marco Cespi, Evelina Serri, Valeria Polzonetti, Adolfo Maria Tambella, Silvia Vincenzetti

**Affiliations:** 1 School of Pharmacy, University of Camerino, 62032 Camerino (MC), Italy; diego.perinelli@unicam.it (D.R.P.); giulia.bonacucina@unicam.it (G.B.); marco.cespi@unicam.it (M.C.); 2 School of Biosciences and Veterinary Medicine, University of Camerino, 62032 Camerino (MC), Italy; stefania.pucciarelli@unicam.it (S.P.); evelina.serri@unicam.it (E.S.); valeria.polzonetti@unicam.it (V.P.); silvia.vincenzetti@unicam.it (S.V.)

**Keywords:** hemocomponent, thrombin-rich solution, storage stability, PRP gels, viscoelastic properties, ELISA test

## Abstract

Platelet-rich plasma (PRP) is a nontransfusional hemocomponent, considered as a powerful concentrate of growth factors (GFs) therapeutically used to stimulate tissue regeneration. The use of autologous PRP, as the patient’s own biological material, for therapeutic purposes represents a safe and effective alternative to conventional treatments in both human and veterinary medicine. The aim of this study was the characterization of canine PRP from rheological and biological points of view. Thus, a characterization of the viscoelastic properties of the PRP systems was performed in order to clarify the influence of different calcium concentrations, in the presence of autologous thrombin-rich solution, on the PRP gels’ mechanical properties, from which the applicability of these systems in biomedical treatments is strongly dependent. Then, an evaluation of the content of GFs in PRP, activated or not with thrombin, and stored at different temperatures (37 °C and −20 °C) was performed over time, outlining, for the first time, the importance of the effect of physiological temperature (37 °C) on the production of GFs. A clinical case study conducted in a dog with a complete rupture of the common calcaneal tendon (Achilles tendon) confirmed the relevance of this hemocomponent in the daily veterinary clinical activity and the potential translational value for human health.

## 1. Introduction

**Figure 1:**
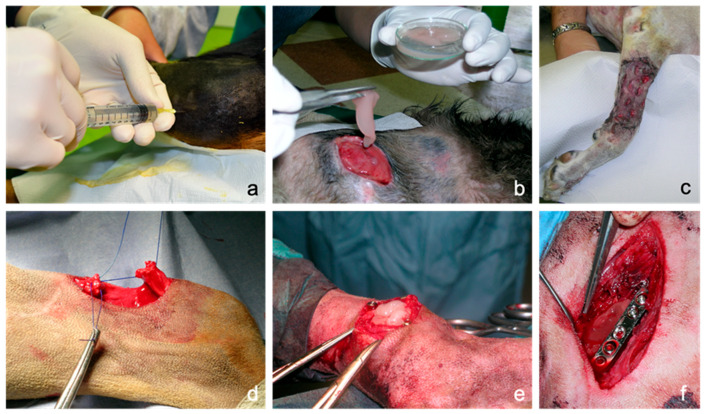
Possible applications of canine platelet-rich plasma (PRP) in the clinical practice: (**a**): intra-articular injection of liquid PRP in a stifle joint to treat osteoarthritis. (**b**): Topical application of PRP in gel formulation in a chronic difficult-to-heal wound over the point of the shoulder. (**c**): Application of PRP for wound bed preparation and engraftment stimulation of a free mesh skin autograft performed in a forelimb traumatic wound with wide loss of substance. (**d**): Use of PRP as biological augmentation for acute common calcaneal tendon (Achilles tendon) rupture surgical repair by end-to-end suture. (**e**): Use of PRP as an alternative to cancellous bone during arthrodesis of a metatarsophalangeal joint. (**f**): Application of PRP during the surgical treatment of a non-union femoral fracture.

Platelet-rich plasma (PRP) is a nontransfusional hemocomponent, which can be therapeutically used as an autologous source of thrombocyte-derived growth factors (GFs) to promote wound healing and tissue repairing for several clinical applications [1]. It can be administered topically in an inactivated liquid form through infiltration or injection for the treatment of skin, joint or tendon disorders. In this case, GFs are released on the application site after platelet activation by endogenous substances such as collagen [2]. Alternatively, PRP can be placed in situ on the affected site after inducing the formation of a clotted/gelatinous material, referred as platelet or PRP gel. PRP gel can be prepared via exogenous activators, as chitosan [3], bovine thrombin [4,5,6,7], thrombin receptor activating peptide (TRAP) [7], ITA gelling agent (Natrex Technologies Inc, Greenville, NC) [4], batroxobin [8], ascorbic acid [9], pulse electric field [7], autologous thrombin [6,10], thrombin/calcium chloride [4], or their combination. These activators are able to induce platelet degranulation and release of GFs, chemokines and other bioactive metabolites, occurring together with the clotting and gelation of the starting PRP. Specifically, the degranulation of platelet granules causes the release of several GFs, including: Platelet-Derived Growth Factor (PDGF), Transforming Growth Factor β (TGF-β), Vascular Endothelial Growth Factor (VEGF), Basic Fibroblast Growth Factor (bFGF), Platelet-Derived Epidermal Growth Factor (PDEGF), Insulin-like Growth Factor-I/II (IGF-I/II) and other active compounds, which are demonstrated to promote regenerative processes including fibroblast chemotaxis, proliferation and differentiation, angiogenesis and epithelial regeneration [11,12,13]. PRP gels have some therapeutically advantages over the liquid form, when applied on skin surfaces, mucous membranes, or in internal tissues during surgery. Specifically, PRP gel has more adhesiveness with respect to liquid PRP, which facilitates its applicability (for instance in the case of wounds) and increases the residence time of the formulation on the injuries [14,15,16]. Moreover, PRP gels have an internal fibrin framework structure, acting as a support regenerative matrix, in which GFs are embedded and slowly released at the injured site [17]. The use of autologous PRP for tissue healing and therapeutic purposes offers a safe and effective alternative to conventional treatments, without relevant side effects being patient’s own biological material [18]. In recent years, PRP gels have been used in vivo for many disorders related to healing process in wounds [19,20,21,22,23,24,25] and musculoskeletal lesions [18,26,27,28,29] both in human and veterinary medicine (Figure 1). Biological (e.g., release of the active compounds) and mechanical (plasticity, modelling and resistance at the level of application site) properties of PRP gels are markedly influenced by platelet concentration and the nature of the activator. Although human autologous PRP gels have been extensively investigated in terms of GFs release [4,5,8,30,31,32], rheological and mechanical properties [33,34,35,36] as well as clinical applications [23,37], are aspects only partially investigated in canine PRP gels. For instance, few studies have reported the GF content of canine PRP gels [38,39,40], while, to the best of the authors’ knowledge, no rheological/mechanical studies were performed on canine PRP gels. The aim of this study was to characterize canine PRP from rheological and biological points of view. The influence of different calcium concentrations together with autologous thrombin-rich solution on the rheological behaviour of the PRP has been assessed. In terms of biological properties, the specific purpose was to evaluate the effect of thrombin activation and storage conditions (37 °C and −20 °C), on the GFs content in PRP over time. Furthermore, in order to frame the results of this study on practical therapeutic aspects, a preliminary case study from daily veterinary clinical activity has been presented.

## 2. Materials and Methods

### 2.1. Blood Collection and PRP Preparation

The study was performed complying with opinion of the committee in charge of animal welfare (OPBA, University of Camerino, protocol number E81AC.10/A) according to the National Legislative Decree 4 March 2014 n. 26, implementation of EU Directive 2010/63/EU. Blood was collected from ten healthy owned dogs at the Veterinary Teaching Hospital, School of Biosciences and Veterinary Medicine, University of Camerino. Informed owner consent for inclusion in the study was obtained for each dog. All dogs were between one and 10 years of age and had a minimum body weight of 15 kg. They had no noteworthy medical problems or medical history, except for osteoarthritis, and were not allowed to be taking any medications other than nutritional or nutraceutical supplements such as glucosamine and chondroitin-sulphate. Upon admission, each dog had a complete physical examination, blood count, and serum biochemical profile. In all dogs, an articular infiltration of autologous PRP was practiced for osteoarthritis management; an aliquot of PRP in excess of the amount needed for infiltration was used for the in vitro investigations of the present study. Blood collection and PRP preparation was performed as described by Tambella and colleagues [24]. Autologous venous blood (50 mL) was withdrawn from the jugular vein of each dog using a 60 mL syringe containing anticoagulant citrate dextrose solution A (ACD-A, Salf S.p.A., Canate Sotto, Bergamo, Italy) at a ratio of 1:9. PRP was prepared by a double spin technique (180 g for 20 min and 650 g for 15 min) for density separation of blood components. Platelet count from whole blood and PRP was performed (Cell Dyn 3500R, Abbott, Wiesbaden, Germany). The platelet concentration in the whole blood ranged from 195 to 335 × 10^3^/μL, while in the PRP it ranged from 907 to 1341 × 10^3^/μL (platelet concentration increased from 3.5- to 5.0-fold). In order to prepare autologous thrombin-rich solution, additional 10 mL of whole blood were collected in two 5 mL sodium citrate tubes (3.8%; Vacuette Tube, Greiner Bio-One GmbH, Kremsmünster, Austria), which were centrifuged at 650× *g* for 10 min. The supernatant plasma fraction was collected in a tube and mixed with calcium gluconate 10% (446 mEq/L of Ca^2+^, Monico SpA, Mestre, Venezia, Italy) at the ratio of 5:1 and then it was incubated at 37 °C for 30 min. The clot obtained was squeezed against the walls of the tube with a sterile Pasteur pipette and the resulting effluent, the thrombin-rich solution, was collected.

### 2.2. PRP Gels Preparation and Rheological Characterization

PRP used for the rheological characterization was stored at −20 °C and, then, thawed prior to use. All PRP gels for the rheological characterization were prepared by mixing directly on the plate of the rotational rheometer (Kinexus Lab+, Malvern Instruments Ltd., Malvern, UK) 1.4 mL of PRP, 0.166 mL of autologous thrombin-rich solution and 0.058 mL of CaCl_2_ (Carlo Erba, Rodano, Italy) solution at three concentrations (216 mM; 432 mM or 648 mM). The concentration of CaCl_2_ in the final formulations were 7.7 mM, 15.4 mM and 23.1 mM, in the range of those reported in the literature [41,42]. Controls without autologous thrombin-rich solution or CaCl_2_ solution were also prepared by adding the corresponding volume of water. PRP gels were prepared directly onto the plate of the rheometer to avoid any eventual breakage of their texture due to the manipulation and to mimic the formation of the gel at the site of application in in vivo experiments. The following rheological tests were performed: (i) stress sweep test at 25 °C and 1Hz in the range 0.1–500 Pa to test the resistance of the gel to mechanical solicitation and breakage/recovery conditions. (ii) Frequency sweep test at 25 °C and a stress of 0.5 Pa to investigate the rheological behaviour of the gels in the range of frequencies 0.01–10 Hz. (iii) All tests were preceded by a time sweep test at 25 °C, 0.5 Pa and 1 Hz for 40 min to assess the rheological stability over time. To assess the influence of storage conditions on the rheological properties of PRP gels, stress and frequency tests were also performed on gels prepared using PRP stored at 4 °C after thawing for 1, 5, 9 and 20 days. 

### 2.3. Determination of Growth Factors Content in PRP

A series of Enzyme-Linked Immunosorbent Assay (ELISA) tests was used to determine the content of GFs in freshly-prepared PRP samples activated (PRPT) or not activated with thrombin (PRPN). The activation was achieved by mixing PRP with thrombin-rich solution at a volumetric ratio 8:1. The determination has been performed on freshly prepared PRPT and PRPN samples (T0) and after incubation for 24 (T24), 48 (T48) and 72 (T72) hours at 37 °C. GFs content was also determined on not-activated frozen-thawed PRPN samples (PRPN-FT). In this case, an aliquot of the freshly-prepared PRPN sample was placed at −20 °C, thawed after 24 h, and the GFs concentration was determined. The ELISA kit used for the growth factors determination were Nori^®^ canine Epidermal Growth Factor (EGF); Nori^®^ canine Fibroblast Growth Factor (FGF); Nori^®^ canine Transforming Growth Factor-beta 2 (TGFβ2); Nori^®^ canine (Platelet-Derived Growth Factor-AA (PDGF-AA); Nori^®^ canine PDGF-AB; Nori^®^ canine PDGF-BB; Nori^®^ canine Vascular Endothelial Growth Factor (VEGF). All ELISA kits were from Genorise Scientific, INC. (Glen Mill, PA, USA). These assays have been performed following the protocol indicated on the datasheet. Briefly, this test is a quantitative sandwich enzyme immunoassay that uses specific antibodies for each growth factor, precoated onto a microplate. In general, samples of 100 μL were applied (eight replicated) to the precoated 96-well plate, subsequently 100 μL of biotinylated polyclonal antibodies were added and incubated for 1h at room temperature. Afterwards, 100 μL avidin-HRP-conjugate was added and incubated for 20 min at room temperature, and finally after an intensive wash, a substrate was added to the wells. The final colour development is proportional to the amount of growth factor bound in the initial step. After the addition of a stop solution, the colour intensity was measured spectrophotometrically at a wavelength of 450 nm using a microplate reader (Multiskan Ascent 96/384 Plate Reader, Dasit, Cornaredo, Italy). To correct the optical imperfections of the plate, readings at 540 nm were subtracted from the reading at 450 nm. For each growth factor determination, a standard curve was generated using as standards the growth factor provided by each kit (EGF: 8–500 pg/mL; FGF: 15–1000 pg/mL; TGFβ2: 28–1800 pg/mL; PDGF-AA: 22–1400 pg/mL; PDGF-AB: 16–1000 pg/mL; PDGF-BB: 28–1800 pg/mL; VEGF: 37–2400 pg/mL). In this case, the standard curve was realized by plotting the mean absorbance for each standard on the y-axis against the concentration on the x-axis. The data were linearized by plotting the log of each standard growth factor concentration versus the log of the absorbance value and the best fitting line was determined by regression analysis. The concentrations of GFs were normalized to 1000 × 10^3^/μL platelets number in all final calculations [43]. Statistical analysis was performed using GraphPad Prism^®^ 6.01 software (GraphPad Software Inc., San Diego, CA, USA). Data were expressed as mean ± SD. The significance of differences among the average content of GFs was determined using two-way ANOVA with Tukey correction employed for multiple comparison. Significant differences between means were indicated when *P* < 0.05.

### 2.4. Application of PRP in Veterinary Medicine

A case study about the potential clinical application of PRP-gel is reported. PRP-gel was used in a dog with complete rupture of the common calcaneal tendon (Achilles tendon) following a direct sharp trauma. At presentation, the dog showed a deep wound in the caudo-lateral aspect of the distal diaphyseal region of the tibia with sudden onset of severe lameness. The dog completely spared the load of the limb in standing position. During weight bearing, the tarsocrural joint sinks in a hyperflex position, with associated stifle hyperextension and plantigrade stance, the typical attitude of complete rupture of the common calcaneal tendon. Surgical therapy was undertaken with approximation of the tendon ends by an end-to-end suture using monofilament material. Autologous PRP gel was positioned as a sleeve around the tendon lesion to obtain a biological augmentation for tendon repair. In order to protect tendon repairing during the initial stages of healing, a bilateral-uniplanar, minimal type, transarticular external skeletal fixator was applied in double frame (calcaneotibial and metatarsotibial) to achieve temporary immobilization of the tarsus. The external fixator was removed four weeks after surgery. An eight-week follow-up was performed.

## 3. Results and Discussion

### 3.1. Rheological Properties of PRP

**Figure 2:**
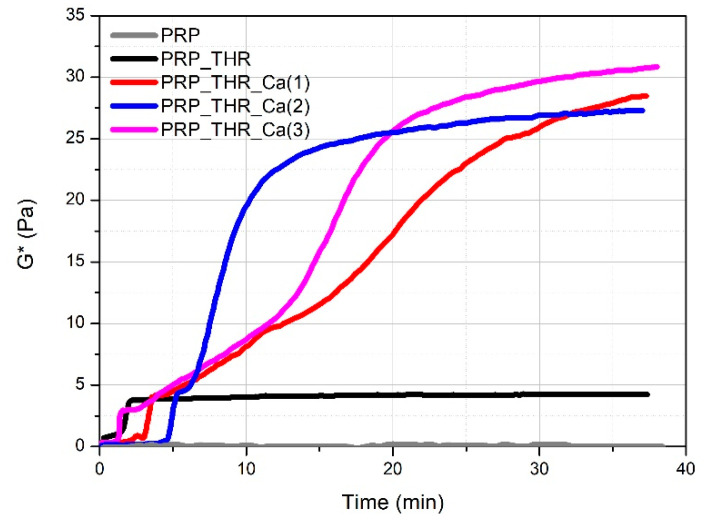
Variation of the complex modulus (G*) over time for PRP alone (PRP), PRP after activation with thrombin (PRP_THR) and in the presence of Ca^2+^ at the three tested concentrations [PRP_THR_Ca(1), 7.7 mM; PRP_THR_Ca(2), 15.4 mM; PRP_THR_Ca(3), 23.1 mM] from time sweep tests at 25 °C.

Mechanical properties are fundamental to investigate in order to evaluate the applicability of PRP gels in veterinary biomedical treatments (e.g., surgery, wound healing). The most commonly applied technique for this purpose is rheology. Rheological measurements have been already performed on PRP gels prepared from human plasma [44,45] but never applied on PRP gels prepared from canine plasma. It is known that a clotting process occurs when platelets are activated by contact of an external activator, which is collagen type 1 in vivo, while a large choice of activators can be used ex vivo. Among them, the most employed method to obtain a clotted material, known as PRP gel, is the activation with thrombin (autologous or synthetic) alone or in presence of Ca^2+^ [41]. The activation involves the degranulation of platelets with a consequent release of GFs, useful for the tissue repairing process and the cleavage of fibrinogen in fibrin, leading to the formation of the matrix in which the GFs are embedded, and responsible of the formation of a clotted “gel-like” material that can be applied for medical treatments. Generally, a soft “gel” with suitable characteristics to be managed and applied during surgery has been obtained by the association of thrombin with Ca^2+^ [24,46]. However, the real effect of thrombin and Ca^2+^ on the consistency of these gels has never been investigated for both human and canine PRP. Consistency of PRP gels can be described from a rheological point of view by the complex modulus G*, keeping into account the contribution of the storage modulus (G′) and the loss modulus (G″) recorded during oscillatory measurements. By following the variation of G* over time (time sweep test, Figure 2), the time needed for the formation of the internal network leading to the gel-like system can be visualised. PRP alone behaves as a liquid material, being the measured G* close to zero. For the other samples, the variation of G* over time shows characteristic steps. A first increase in the consistency occurs in the first 5 min from mixing the components (PRP, thrombin-rich solution and Ca^2+^), which can be attributed to the early effect of thrombin. This time lapse, referring to the conversion of fibrinogen in fibrin, is comparable to that already reported for the clotting of PRP with autologous thrombin [47]. The adding of Ca^2+^ determines a further remarkable increase in consistency, which is four- to fivefold higher than the sample activated with the only thrombin-rich solution. On the other side, the time for clotting (between 2.5 min and 6 min) as well as for reaching the final consistency becomes longer by up to 30–40 min. Indeed, in the presence of Ca^2+^, the rheological properties of PRP gels are predominantly affected by the cation rather than by thrombin. This means that Ca^2+^ is able to promote the internal network structure of PRP gels, by increasing the rate of fibrin monomer polymerization [48] or the physical cross-linking between fibrin peptides favoured by the activation of XIII coagulation factor [49]. Among the tested Ca^2+^ concentrations, the gel prepared with 15.4 mM of Ca^2+^ solution showed a good compromise in terms of maximum consistency (G* ~27 Pa) and time for reaching G* plateau value (around 25 min).

**Figure 3:**
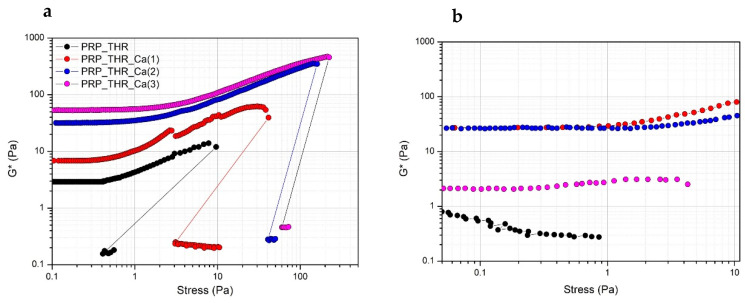
Variation of the complex modulus (G*) over the applied stress (Pa) for PRP after activation with thrombin (PRP_THR) and in presence of Ca^2+^ at the three tested concentrations [PRP_THR_Ca(1), 7.7 mM; PRP_THR_Ca(2) 15.4 mM; PRP_THR_Ca(3), 23.1 mM]. Two consecutive stress sweep tests were performed at 25 °C to highlight the breakage point of PRP formulations (**a**) and their recovery (**b**).

In terms of resistance to the applied mechanical solicitation (tangential stress), all PRP gels show the typical strain hardening effect, as widely described in the literature [45,50]. Indeed, PRP gels, as well as many filamentous biopolymer dispersions (such as actin, collagen, fibrin) have a typical nonlinear viscoelastic mechanical response when deformed in shear, resulting in a stiffing or hardening effect on the systems over the applied stresses. This effect occurs at relatively low stress values, at which the large majority of polymeric dispersions behaves differently. Generally, for most of the polymeric dispersions, the rheological moduli are independent of the applied stress (linear viscoelastic regimen), then they show a decrease of the stress values at which the internal structure of the samples collapses. The effect of strain hardening can be explained by the semiflexible nature of the fibrin filaments, composing PRP gels [51,52]. Fibrin filaments show a nonlinear force extension relation, probably due to an increased elastic response of the polymeric network. This increased elasticity results from a larger steric contact at the filament network junction, when deformed in shear, due to possible collective rearrangements or alignments of fibrin filaments at a microscopic scale [53,54]. The stiffening is clearly visible above a stress of 1 Pa for all systems, while below this stress value the mechanical solicitation is possibly not sufficient to induce this rheological effect. The measured G* value still increases up to a critical value, at which G* modulus abruptly drops, indicating the collapse of the microstructure. At this stress value, the internal networking structure is lost and samples become liquid-like, as revealed by the much lower G* values (around 0.1 Pa) recorded just after the brakeage. The structural effect of Ca^2+^ on PRP gels can be also highlighted. Indeed, the measured G* values are higher for systems prepared with a larger amount of Ca^2+^ in all range of applied stresses, with the major effect observable when a 15.4 mM of Ca^2+^ is added. A further increase of Ca^2+^ exerts only a negligible effect on G* value. The effect of Ca^2+^ is also evident by looking at the critical stress values. These values are higher for increasing Ca^2+^ concentrations in the systems. Particularly, these values are around 40 Pa for the system prepared with a 7.7 mM of Ca^2+^ and increase up to 100 Pa for the system with 15.4 mM and up to 250 Pa for the system prepared with a 23.1 mM of Ca^2+^ (Figure 3a). Interestingly, if another stress sweep test is performed after 5 min from the end of the previous one, a certain recovery of the viscoelastic behaviour is observed. In this case, a major recovery was observed for the system prepared by adding 7.7 mM of Ca^2+^, no recovery, instead, was observed for the sample prepared with 23.1 mM of Ca^2+^. Thus, Ca^2+^, while promoting the consistency of PRP gels and increasing the resistance to brakeage at higher applied stresses, also exerted a negative effect on the recovery of the viscoelastic properties of PRP gels (Figure 3b). This can be explained by supposing that the higher critical stress to be applied for gel breakage in the presence of large amount of Ca^2+^ eventually determines an irreversible effect on the cross-linking sites of the fibrin network.

**Figure 4:**
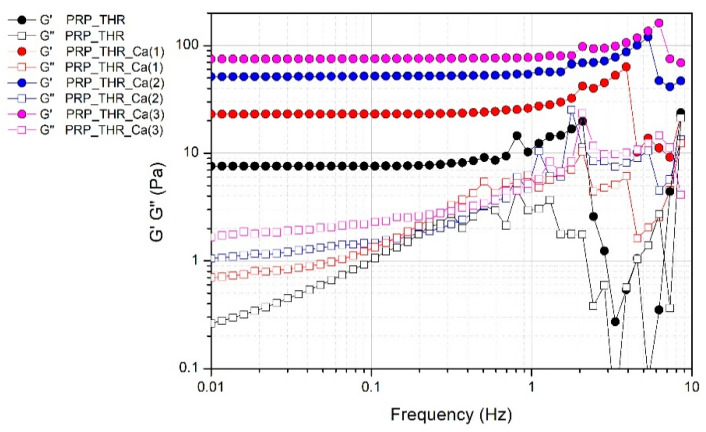
Variation of the elastic modulus (G′) and viscous modulus (G″) over the applied frequency (0.1–10 Hz) for PRP after activation with thrombin (PRP_THR) and in presence of Ca^2+^ at the three tested concentrations [PRP_THR_Ca(1), 7.7 mM; PRP_THR_Ca(2), 15.4 mM; PRP_THR_Ca(3), 23.1 mM] from frequency sweep test at 25 °C.

The presence of a fibrin network in PRP gels is also confirmed by the results of frequency sweep test (Figure 4). Despite the not marked consistency of PRP gels as resulted from the measured G* values, these systems can be considered real gels from a rheological point of view, thereby showing a solid-like behaviour, since the elastic modulus G′ is higher than the viscous modulus G″ in all the range of frequencies analysed. Indeed, G′ modulus is quite independent of the frequency (almost a constant value for G′ in a wide range of frequencies) differently from G″ modulus, which shows an increasing trend at least in the range of frequencies 0.01–2 Hz. The effect of frequency on G″ is particularly evident in the sample without Ca^2+^, while it is less marked on the samples prepared with an increasing amount of Ca^2+^, confirming the role of this cation in promoting the structuring of the PRP gels.

**Figure 5:**
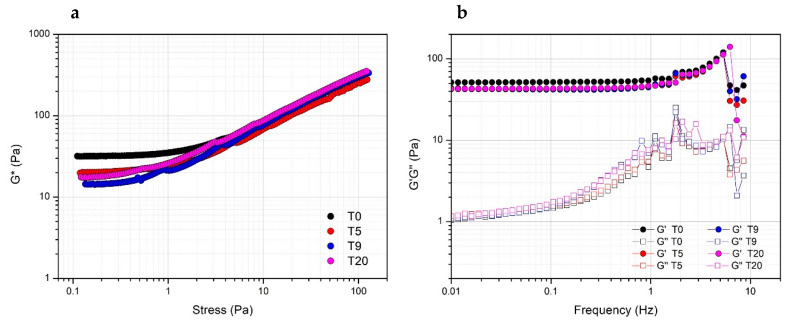
Effect of the storage time at 4 °C of PRP after thawing as evaluated from the rheological properties (complex modulus G*) of PRP_THR_Ca(2) gel from stress sweep test (**a**) and frequency sweep test (elastic modulus G′ and viscous modulus G″) (**b**) at different timepoints (T0, T5, T9, T20 days).

Storage stability of PRP at 4 °C was evaluated by comparing the rheological properties of gels prepared with Ca^2+^ at a concentration of 15.4 mM the same day or after five, nine and 20 days from thawing. Actually, both in terms of resistance to the applied stress (Figure 5a) or viscoelastic properties (Figure 5b), the obtained gels prepared at a different storage time are comparable, indicating the stability of PRP, which can be used for the preparation of the gel for biomedical application at least up to 20 days from the collection.

### 3.2. Quantitative Determination of Growth Factors in PRP

**Figure 6:**
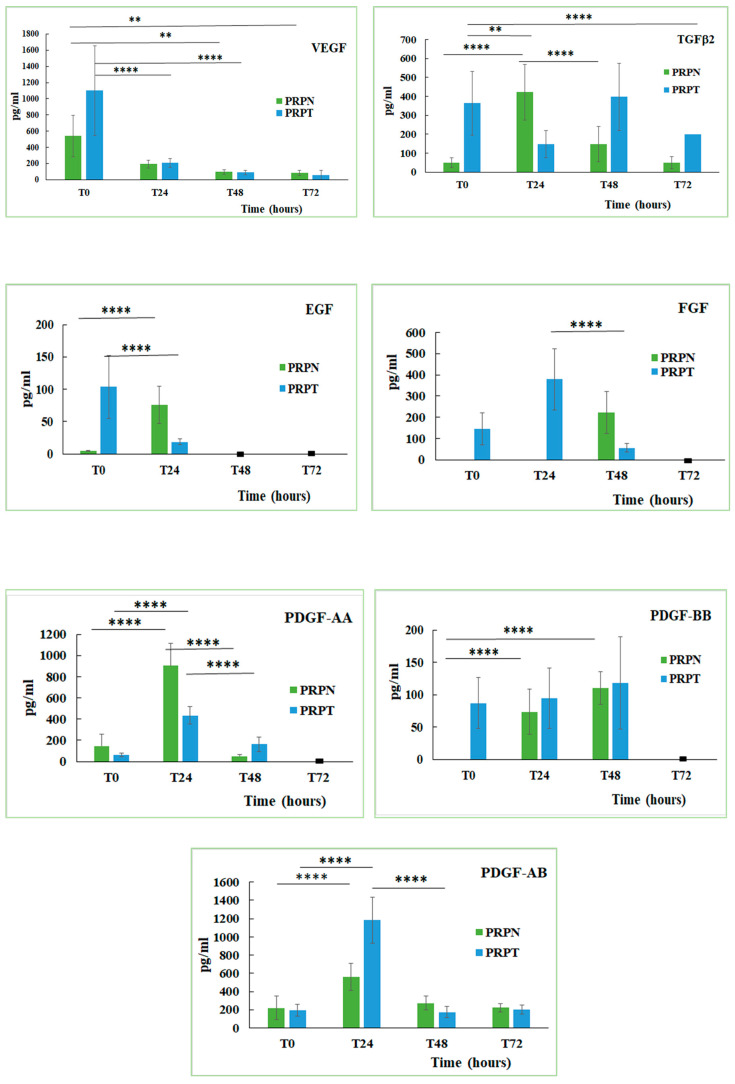
Concentration (pg/mL) of growth factors (Vascular Endothelial Growth Factor, VEGF; Transforming Growth Factor β2, TGFβ2; Endothelial Growth Factor, EGF; Fibroblast Growth Factor, FGF; Platelet-derived Growth Factor-AA, PDGF-AA; Platelet-derived Growth Factor-BB, PDGF-BB; Platelet-derived Growth Factor-AB, PDGF-AB) in PRP activated (PRPT) or not activated (PRPN) with autologous thrombin-rich solution at T0 and after incubation at 37 °C for 24 h (T24), 48 h (T48) and 72 h (T72). ** *P* < 0.01; **** *P* < 0.0001. (■): value lower than the limit of detection.

Platelets represent a reservoir of several growth factors that, once released, may regulate tissue reparative processes. Growth factors are bioactive peptides involved in the regulation of the cell proliferation and differentiation from epithelium, bone and connective tissues [55]. The content of some growth factors in the canine PRP stored at different conditions and activated (PRPT) or not (PRPN) with thrombin has been determined. Here below, the equations of the calibration lines for each GF, with the corresponding correlation coefficient (R^2^) values are shown: EGF y = 0.024x + 0.0375, R^2^ = 0.996; FGF y = 0.0074x + 0.0091, R^2^ = 0.971; TGFβ2 y = 0.0003x + 0.1195, R^2^ = 0.999; PDGF-AA y = 0.0013x + 0.0456, R^2^ = 0.995; PDGF-AB y = 0.0002x + 0.0048, R^2^ = 0.913; PDGF-BB y = 0.0003x + 0.0345, R^2^ = 0.986; VEGF y = 0.0005x + 0.0011, R^2^ = 0.950. The function of thrombin, a multifunctional protease, is to activate the haemostasis and the coagulation stimulating the fibrin deposition and therefore promoting platelet aggregation. Thrombin is also involved in the connective tissue remodelling processes, in both normal tissue and vascular repair, in proinflammatory processes and in several pathologic conditions associated with the activation of the coagulation cascade [56]. The obtained results indicated that each growth factor behaved differently depending on whether the PRP is activated or not with thrombin (Figure 6). The freezing-thawing process also exerts a different effect on the content of each growth factor (Table 1). In general, the activation with thrombin leads to a greater release of the GFs in the freshly prepared PRP samples, while the not-activated sample tends to increase their concentration only after the incubation at 37 °C for several hours. By analysing the behaviour of each growth factor examined in this study, it can be seen that in the freshly prepared PRP the VEGF concentration is double in the thrombin-activated samples compared to those not activated (PRPN-T0: 541.3 ± 255.4 pg/mL; PRPT-T0: 1100.2 ± 550.3 pg/mL). Incubation of both PRP samples (activated and not activated) for 24, 48 and 72 h at 37 °C, led to a drastic decrease of VEGF concentration (PRPT-T0: 1100.2 ± 550.3 pg/mL; PRPT-T24: 209 ± 53.9 pg/mL, *P* < 0.0001). Furthermore, the freezing-thawing procedure affected significantly VEGF concentration in the PRP sample (PRPN-T0: 541.3 ± 255.4 pg/mL; PRPN-FT: 114.8 ± 44.2 pg/mL; *P* < 0.05). It is known that VEGF stimulates chemotaxis and proliferation of endothelial cells in order to promote vasculogenesis and angiogenesis. It also raises the hyperpermeability of blood vessels [12,57]. In addition, in the case of TGFβ2, the thrombin activation leads to a higher concentration of this GF with respect to the not-activated counterpart (PRPN-T0: 48.8 ± 25.1 pg/mL; PRPT-T0: 363.9 ± 167.8 pg/mL). The concentration of TGFβ2, reached the highest value in the PRPT sample, after 48 h incubation at 37 °C (PRP-T48: 397.2 ± 177.7 pg/mL). In the not-activated PRP, TGFβ2 reached a concentration tenfold higher after 24 h incubation at 37 °C with respect to the freshly prepared sample (PRPN-T0: 48.8 ± 25.1 pg/mL; PRPN-T24: 421.9 ± 145.9 pg/mL; *P* < 0.0001). The process of freezing-thawing did not affect the concentration of TGFβ2. This GF stimulates collagen type II synthesis and proteoglycan synthesis; it has been shown that TGFβ induces chondrogenesis of mesenchymal stem cells derived from bone marrow by deposition of a cartilage-specific extracellular matrix [58]. PDGF is a 30 kDa dimer composed of two main polypeptides (A and B) and two additional polypeptides (C and D), all of them encoded by separate genes. There are different isoforms of PDGF formed by the combination of the polypeptide chains, but only three of them are released in a biologically active form: PDGF-AA, PDGF-BB, PDGF-AB. PDGF is mitogenic for mesenchymal cells such as fibroblasts, osteoblasts and adipocytes, and stimulates the formation of collagen type I [59,60,61]. PDGF is involved in angiogenesis, erythropoiesis, bone formation, wound healing and also in the normal development of the cardiovascular and respiratory systems, brain and kidney [12,62]. In the canine PRP, the isoform PDGF-AA reached a high concentration only after 24 h incubation at 37 °C, independently from thrombin activation (PRPN-T24: 904.1 ± 212.5 pg/mL, PRPT-T24: 433.3 ± 82.7 pg/mL). On the contrary, the isoform PDGF-BB was activated in the freshly-prepared sample only after thrombin activation (PRPN-T0: not detected; PRPT-T0: 86.7 ± 39.4 pg/mL). After 24 h incubation at 37 °C, PDGF-BB was also detected in the not-activated PRP, and after 48 h the concentration resulted comparable to that observed in the activated PRP (PRPN-T48: 110.2 ± 25.3 pg/mL; PRPT-T48: 118.1 ± 71.1 pg/mL). The isoform PDGF-AB behaved in an intermediate manner with the respect to the other two isoforms. In general, the freezing-thawing procedure seems not to affect the PDGF concentration independently from the isoform. EGF concentration is higher in the freshly prepared PRP activated with thrombin (PRPT-T0: 103.53 ± 48.5 pg/mL) with respect to the same not activated sample (PRPN-T0: 4.7 ± 0.9 pg/mL). After 24 h of incubation at 37 °C, EGF concentration increased in the PRPN sample (PRPN-T0: 4.7 ± 0.9 pg/mL; PRPN-T24: 76.1 ± 28.9 pg/mL; *P* < 0.0001). At longer durations (48 and 72 h), instead, EGF was not detected. In the PRPT samples, EGF concentration decreased significantly during the first 24 h of incubation at 37 °C (PRPT-T0: 103.53 ± 48.5 pg/mL; PRPT-T24: 18.6 ± 4.2 pg/mL; *P* < 0.0001). PRPN freezing-thawing procedure leads to a complete loss of the EGF concentration. Some authors showed that EGF, released at high concentration in human PRP supernatants, is an important activator of endothelial cells in vitro and for this reason they suggested the use of PRP for the regeneration of the periodontal tissue [54]. Regarding the behaviour of fibroblast growth factor (FGF), it can be seen that its concentration is detectable in the PRP samples only after thrombin activation. In fact, this GF resulted completely absent in the PRPN samples freshly prepared (PRPN-T0) and after 24 h incubation at 37 °C (PRPN-T24), appearing only after 48 h of incubation at 37 °C. In the presence of thrombin, FGF is released at T0 (146.6 ± 75.5 pg/mL), reaching the highest concentration value after 24 h incubation at 37 °C (378.9 ± 145 pg/mL). In this case, it was not possible to analyse the effect of freezing-thawing on FGF, since this growth factor was not detectable at T0. FGF has effect on tissues repair and regeneration such as muscle, skin, tendon/ligament, cartilage, blood vessel, adipose, bone, tooth, and nerve. FGF has a role in mitogenesis, differentiation, angiogenesis, cellular migration, and also wound healing. Recently, the effects of storage conditions on GF release of human PRP have been studied [63,64,65]. All these works have highlighted the importance of the storage time and temperature on GFs release from PRP and how these parameters can be controlled to modulate its biological properties. However, no previous studies have investigated the effect of physiological temperature (37 °C) on the concentration of GFs in PRP at different times. In general, the storage at 37 °C has determined a decrease in GFs concentrations after 24 h in PRP both activated or not with thrombin. Moreover, the freezing-thawing procedure of the prepared PRP samples exerts also an effect on GFs content, leading to a decrease in concentration of VEGF, EGF, PDGF-AB. Similarly, other authors [63,66] found that the levels of several GFs (e.g. EGF, VEGF, FGF, PDGF, IGF-1, and TGFβ1) were significantly decreased after storing PRP at –20 °C and a freezing-thawing process.

### 3.3. Application of PRP Gel in Veterinary Medicine

**Figure 7:**
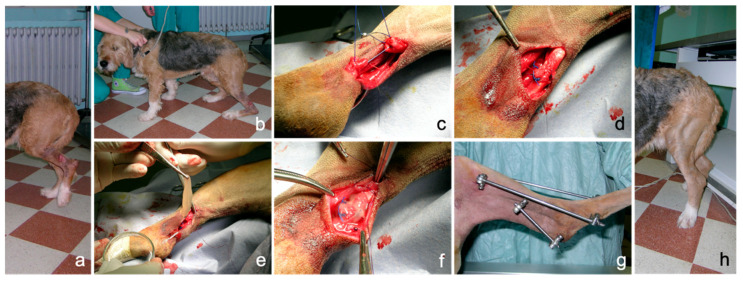
Complete rupture of the common calcaneal tendon (Achille tendon) in a dog following direct sharp trauma. (**a**): condition at presentation showing a deep wound at the tibial diaphyseal region and complete hind limb load sparing in standing position; (**b**): plantigrade posture during weight bearing; (**c**): surgical approximation of the tendon ends; (**d**): completed suture pattern; (**e**): autologous PRP-gel application; (**f**): PRP-gel positioned as a sleeve around the tendon lesion; (**g**): transarticular external skeletal fixation; (**h**): correct limb support eight weeks after surgery.

No technical problems occurred during application; the properties of consistency, elasticity and adherence of the PRP gel allowed an easy application as a sleeve around the sutured common calcaneal tendon. PRP gel was well-tolerated without apparent side effects. In this case, lameness and weight load on the injured limb improved progressively during follow-up, both during the permanence of the external fixator and after its removal, until a correct hind limb support was obtained. An overall iconographic documentation is reported in Figure 7, summarizing the initial condition, the surgical treatment, the intraoperative application of PRP and the positive outcome achieved in this clinical case (Figure 7).

Treatment of complete rupture of the common calcaneal tendon is not an easy-to-treat injury in human beings, but it is particularly challenging in dogs, also considering the anatomo-physiological and biomechanical similarities and differences between people and dogs. As suggested in other studies [66,67], PRP could be used as a biological augmentation for surgical repair of the acute common calcaneal tendon as it is potentially able to improve functional outcomes.

## 4. Conclusions

In this study, rheological and biological aspects of canine PRP have been characterized in order to rationalize its use in veterinary biomedical treatments. In particular, the mechanical properties of PRP gels obtained by adding different calcium concentrations as activator in the presence of thrombin were investigated by rheology. The monitoring of the different rheological parameters clearly demonstrated that Ca^2+^ is able to promote the internal network structure of PRP gels making possible the obtainment of structured systems with viscoelastic properties and stability, suitable for a potential application. Furthermore, the effect of physiological temperature (37 °C) on the production of GFs from the PRP at different times has been studied. The content determination of GFs in PRP (activated or not with thrombin) confirmed that GFs production is dependent on the storage conditions making possible the modulation of a specific GF concentration, by controlling storage time and temperature (37 °C and −20 °C). Thus, this study confirmed that the GFs concentration in PRP preparations is variable and strictly depends on the storage temperature and duration. Furthermore, the results obtained in the clinical case study, reported as an example of PRP gels’ applicability, confirmed the potentiality of this hemocomponent in the daily veterinary clinical activity. Indeed, this study could set the stage for the development of in vivo studies using canine animal models, which assure a more reliable assessing of the clinical efficacy of PRP compared to experimentally induced injuries. In this context, the results of this characterization study on PRP could have a value for the canine species diseases itself and could also have a high translational value for human health.

## Figures and Tables

**Table 1:** Concentration of growth factors (pg/mL) in fresh not-activated PRP samples (PRPN-T0) and not-activated PRP samples frozen-thawed (PRPN-FT).

Growth Factor	PRPN-T0 (pg/mL)	PRPN-FT (pg/mL)
VEGF	541.3 ± 255.4 ^a^	114.8 ± 44.2 ^b^
TGFβ-2	48.8 ± 25.1 ^a^	254.8 ± 102.2 ^c^
EGF	4.7 ± 0.9	N.D.
FGF	N.D.	N.D.
PDGF-AA	146.9 ± 108.4	155.5 ± 68.5
PDGF-BB	N.D.	80.3 ± 33.4
PDGF-AB	218.9 ± 130.6	154.9 ± 84.9

Different letters on the same row indicate a statistically significant difference (a, b: *P* < 0.05; a, c: *P* < 0.01). N.D. = Not Detected

## References

[1] Tambella A.M., Martin S., Cantalamessa A., Serri E., Attili A.R. (2018). Platelet-rich Plasma and Other Hemocomponents in Veterinary Regenerative Medicine. Wounds Compend. Clin. Res. Pract..

[2] Farndale R.W. (2006). Collagen-induced platelet activation. Blood Cells. Mol. Dis..

[3] Shen E.-C., Chou T.-C., Gau C.-H., Tu H.-P., Chen Y.-T., Fu E. (2006). Releasing growth factors from activated human platelets after chitosan stimulation: A possible bio-material for platelet-rich plasma preparation. Clin. Oral Implants Res..

[4] Landesberg R., Roy M., Glickman R.S. (2000). Quantification of growth factor levels using a simplified method of platelet-rich plasma gel preparation. J. Oral Maxillofac. Surg..

[5] Martineau I., Lacoste E., Gagnon G. (2004). Effects of Calcium and Thrombin on Growth Factor Release from Platelet Concentrates: Kinetics and Regulation of Endothelial Cell Proliferation. Biomaterials.

[6] Semple E., Speck E., Aslam R., Kim M., Kumar V., Semple J. (2008). Evaluation of Platelet Gel Characteristics Using Thrombin Produced by the Thrombin Processing Device: A Comparative Study. J. Oral Maxillofac. Surg..

[7] Frelinger A.L., Torres A.S., Caiafa A., Morton C.A., Berny-Lang M.A., Gerrits A.J., Carmichael S.L., Neculaes V.B., Michelson A.D. (2016). Platelet-rich plasma stimulated by pulse electric fields: Platelet activation, procoagulant markers, growth factor release and cell proliferation. Platelets.

[8] Rughetti A., Gallo R., Caloprisco G., Borean A., Necozione S., Dell’Orso L., Leocata P., Rivellini C., Di Marzio D., Di Natale D. (2006). Platelet gel: Assays of three growth factors. Blood Transfus.

[9] Carter C.A., Jolly D.G., Worden C.E., Hendren D.G., Kane C.J.M. (2003). Platelet-rich plasma gel promotes differentiation and regeneration during equine wound healing. Exp. Mol. Pathol..

[10] Crovetti G., Martinelli G., Issi M., Barone M., Guizzardi M., Campanati B., Moroni M., Carabelli A. (2004). Platelet gel for healing cutaneous chronic wounds. Transfus. Apher. Sci..

[11] Mazzucco L., Borzini P., Gope R. (2010). Platelet-derived Factors Involved in Tissue Repair-From Signal to Function. Transfus. Med. Rev..

[12] Anitua E., Alkhraisat M.H., Orive G. (2012). Perspectives and challenges in regenerative medicine using plasma rich in growth factors. J. Control. Release.

[13] Marques L.F., Stessuk T., Camargo I.C.C., Sabeh Junior N., dos Santos L., Ribeiro-Paes J.T. (2015). Platelet-rich plasma (PRP): Methodological aspects and clinical applications. Platelets.

[14] Giraldo C., López C., Álvarez M., Samudio I., Prades M., Carmona J. (2013). Effects of the Breed, Sex and Age on Cellular Content and Growth Factor Release From Equine Pure-Platelet Rich Plasma and Pure-Platelet Rich Gel. BMC Vet. Res..

[15] Karayannopoulou M., Papazoglou L., Loukopoulos P., Kazakos G., Chantes A., Giannakas N., Savvas I., Psalla D., Kritsepi-Konstantinou M., Dionyssiou D. (2014). Locally Injected Autologous Platelet-Rich Plasma Enhanced Tissue Perfusion and Improved Survival of Long Subdermal Plexus Skin Flaps in Dogs. Vet. Comp. Orthop. Traumatol..

[16] Woo S.H., Jeong H.-S., Kim J.P., Koh E.-H., Lee S.U., Jin S.M., Kim D.H., Sohn J.H., Lee S.H. (2014). Favorable Vocal Fold Wound Healing Induced by Platelet-Rich Plasma Injection. Clin. Exp. Otorhinolaryngol..

[17] Anitua E., Nurden P., Prado P., Nurden A., Padilla S. (2019). Autologous Fibrin Scaffolds: When Platelet- And Plasma-Derived Biomolecules Meet Fibrin. Biomaterials.

[18] Brossi P.M., Moreira J.J., Machado T.S.L., Baccarin R.Y.A. (2015). Platelet-rich plasma in orthopedic therapy: A comparative systematic review of clinical and experimental data in equine and human musculoskeletal lesions. BMC Vet. Res..

[19] Kim J., Park C., Park H. (2009). Curative Effect of Autologous Platelet-Rich Plasma on a Large Cutaneous Lesion in a Dog. Vet. Dermatol..

[20] Burgos-Alonso N., Lobato I., Hernández I., Sebastian K.S., Rodríguez B., March A.G., Perez-Salvador A., Arce V., Garcia-Alvarez A., Gomez-Fernandez M.C. (2018). Autologous platelet-rich plasma in the treatment of venous leg ulcers in primary care: A randomised controlled, pilot study. J. Wound Care.

[21] Tambella A., Attili A., Dupré G., Cantalamessa A., Martin S., Cuteri V., Marcazzan S., Del Fabbro M. (2018). Platelet-rich Plasma to Treat Experimentally-Induced Skin Wounds in Animals: A Systematic Review and Meta-Analysis. PLoS ONE.

[22] Moneib H., Youssef S., Aly D., Rizk M., Abdelhakeem Y. (2018). Autologous Platelet-Rich Plasma Versus Conventional Therapy for the Treatment of Chronic Venous Leg Ulcers: A Comparative Study. J. Cosmet. Dermatol..

[23] Martinez-Zapata M.J., Martí-Carvajal A.J., Solà I., Expósito J.A., Bolíbar I., Rodríguez L., Garcia J., Zaror C. (2016). Autologous platelet-rich plasma for treating chronic wounds. Cochrane Database Syst. Rev..

[24] Tambella A., Attili A., Dini F., Palumbo Piccionello A., Vullo C., Serri E., Scrollavezza P., Dupré G. (2014). Autologous Platelet Gel to Treat Chronic Decubital Ulcers: A Randomized, Blind Controlled Clinical Trial in Dogs. Vet. Surg..

[25] Picard F., Hersant B., Bosc R., Meningaud J.-P. (2015). Should we use platelet-rich plasma as an adjunct therapy to treat “acute wounds”, “burns”, and “laser therapies”: A review and a proposal of a quality criteria checklist for further studies. Wound Repair Regen..

[26] Samy A.M. (2016). The role of platelet rich plasma in management of fracture neck femur: New insights. Int. Orthop..

[27] Faillace V., Tambella A.M., Fratini M., Paggi E., Dini F., Laus F. (2017). Use of autologous platelet-rich plasma for a delayed consolidation of a tibial fracture in a young donkey. J. Vet. Med. Sci..

[28] Marcazzan S., Taschieri S., Weinstein R., Del Fabbro M. (2018). Efficacy of Platelet Concentrates in Bone Healing: A Systematic Review on Animal Studies—Part B: Large-size Animal Models. Platelets.

[29] Huang Y., Liu X., Xu X., Liu J. (2019). Intra-articular injections of platelet-rich plasma, hyaluronic acid or corticosteroids for knee osteoarthritis: A prospective randomized controlled study. Orthopade.

[30] Weibrich G., Kleis W., Hafner G., Hitzler W. (2002). Growth Factor Levels in Platelet-Rich Plasma and Correlations with Donor Age, Sex, and Platelet Count. J. Craniomaxillofac. Surg..

[31] Leitner G.C., Gruber R., Neumüller J., Wagner A., Kloimstein P., Höcker P., Körmöczi G.F., Buchta C. (2006). Platelet content and growth factor release in platelet-rich plasma: A comparison of four different systems. Vox Sang..

[32] McCarrel T., Fortier L. (2009). Temporal growth factor release from platelet-rich plasma, trehalose lyophilized platelets, and bone marrow aspirate and their effect on tendon and ligament gene expression. J. Orthop. Res..

[33] Pallotta I., Kluge J., Moreau J., Calabrese R., Kaplan D., Balduini A. (2014). Characteristics of Platelet Gels Combined with Silk. Biomaterials.

[34] Russo F., D’Este M., Vadalà G., Cattani C., Papalia R., Alini M., Denaro V. (2016). Platelet Rich Plasma and Hyaluronic Acid Blend for the Treatment of Osteoarthritis: Rheological and Biological Evaluation. PLoS ONE.

[35] Ghazouane R., Bertrand B., Philandrianos C., Veran J., Abellan M., Francois P., Velier M., Orneto C., Piccerelle P., Magalon J. (2017). What about the Rheological Properties of PRP/Microfat Mixtures in Fat Grafting Procedure?. Aesthetic Plast. Surg..

[36] Vadalà G., Russo F., Musumeci M., D’Este M., Cattani C., Catanzaro G., Tirindelli M., Lazzari L., Alini M., Giordano R. (2017). Clinically Relevant Hydrogel-Based on Hyaluronic Acid and Platelet Rich Plasma as a Carrier for Mesenchymal Stem Cells: Rheological and Biological Characterization. J. Orthop. Res..

[37] Del Fabbro M., Bucchi C., Lolato A., Corbella S., Testori T., Taschieri S. (2017). Healing of Postextraction Sockets Preserved with Autologous Platelet Concentrates. A Systematic Review and Meta-Analysis. J. Oral Maxillofac. Surg..

[38] Stief M., Gottschalk J., Ionita J.-C., Einspanier A., Oechtering G., Böttcher P. (2011). Concentration of platelets and growth factors in canine autologous conditioned plasma. Vet. Comp. Orthop. Traumatol..

[39] Franklin S., Birdwhistell K. (2018). Assessment of Canine Autologous Conditioned Plasma TM Cellular and Transforming Growth Factor-β1 Content. Front. Vet. Sci..

[40] Shin H.-S., Woo H.-M., Kang B.-J. (2017). Optimisation of a double-centrifugation method for preparation of canine platelet-rich plasma. BMC Vet. Res..

[41] Jalowiec J.M., D’Este M., Bara J.J., Denom J., Menzel U., Alini M., Verrier S., Herrmann M. (2016). An In Vitro Investigation of Platelet-Rich Plasma-Gel as a Cell and Growth Factor Delivery Vehicle for Tissue Engineering. Tissue Eng. Part C. Methods.

[42] Toyoda T., Isobe K., Tsujino T., Koyata Y., Ohyagi F., Watanabe T., Nakamura M., Kitamura Y., Okudera H., Nakata K. (2018). Direct activation of platelets by addition of CaCl2 leads coagulation of platelet-rich plasma. Int. J. Implant Dent..

[43] Weibrich G., Hansen T., Kleis W., Buch R., Hitzler W. (2004). Effect of Platelet Concentration in Platelet-Rich Plasma on Peri-Implant Bone Regeneration. Bone.

[44] Shah J.V., Janmey P.A. (1997). Strain hardening of fibrin gels and plasma clots. Rheol. Acta.

[45] Cavallo C., Roffi A., Grigolo B., Mariani E., Pratelli L., Merli G., Kon E., Marcacci M., Filardo G. (2016). Platelet-Rich Plasma: The Choice of Activation Method Affects the Release of Bioactive Molecules. BioMed Res. Int..

[46] Greppi N., Mazzucco L., Galetti G., Bona F., Petrillo E., Smacchia C., Raspollini E., Cossovich P., Caprioli R., Borzini P. (2011). Treatment of recalcitrant ulcers with allogeneic platelet gel from pooled platelets in aged hypomobile patients. Biologicals.

[47] Huber S.C., Cunha Júnior J.L.R., Montalvão S., da Silva L.Q., Paffaro A.U., da Silva F.A.R., Rodrigues B.L., Lana J.F.S.D., Annichino-Bizzacchi J.M. (2016). In vitro study of the role of thrombin in platelet rich plasma (PRP) preparation: Utility for gel formation and impact in growth factors release. J. Stem Cells Regen. Med..

[48] Brass E., Forman W., Edwards R., Lindan O. (1978). Fibrin formation: Effect of calcium ions. Blood.

[49] Weisel J.W., Litvinov R.I. (2013). Mechanisms of fibrin polymerization and clinical implications. Blood.

[50] Van Oosten A.S.G., Vahabi M., Licup A.J., Sharma A., Galie P.A., MacKintosh F.C., Janmey P.A. (2016). Uncoupling shear and uniaxial elastic moduli of semiflexible biopolymer networks: Compression-softening and stretch-stiffening. Sci. Rep..

[51] Bale M., Ferry J. (1988). Strain Enhancement of Elastic Modulus in Fine Fibrin Clots. Thromb. Res..

[52] Janmey P., Erdile L., Bale M., Ferry J. (1983). Kinetics of Fibrin Oligomer Formation Observed by Electron Microscopy. Biochemistry.

[53] Storm C., Pastore J., MacKintosh F., Lubensky T., Janmey P. (2005). Nonlinear Elasticity in Biological Gels. Nature.

[54] Onck P.R., Koeman T., van Dillen T., van der Giessen E. (2005). Alternative explanation of stiffening in cross-linked semiflexible networks. Phys. Rev. Lett..

[55] Marx R.E., Carlson E.R., Eichstaedt R.M., Schimmele S.R., Strauss J.E., Georgeff K.R. (1998). Platelet-rich Plasma: Growth factor enhancement for bone grafts. Oral Surg. Oral Med. Oral Pathol. Oral Radiol. Endod..

[56] Chambers R., Leoni P., Blanc-Brude O., Wembridge D., Laurent G. (2000). Thrombin Is a Potent Inducer of Connective Tissue Growth Factor Production via Proteolytic Activation of Protease-Activated receptor-1. J. Biol. Chem..

[57] Anitua E., Andia I., Ardanza B., Nurden P., Nurden A.T. (2004). Autologous platelets as a source of proteins for healing and tissue regeneration. Thromb. Haemost..

[58] Barry F., Boynton R., Liu B., Murphy J. (2001). Chondrogenic Differentiation of Mesenchymal Stem Cells from Bone Marrow: Differentiation-Dependent Gene Expression of Matrix Components. Exp. Cell Res..

[59] Hock J., Canalis E. (1994). Platelet-derived Growth Factor Enhances Bone Cell Replication, but Not Differentiated Function of Osteoblasts. Endocrinology.

[60] Chen R.R., Silva E.A., Yuen W.W., Mooney D.J. (2007). Spatio-temporal VEGF and PDGF delivery patterns blood vessel formation and maturation. Pharm. Res..

[61] Kakudo N., Minakata T., Mitsui T., Kushida S., Notodihardjo F., Kusumoto K. (2008). Proliferation-promoting Effect of Platelet-Rich Plasma on Human Adipose-Derived Stem Cells and Human Dermal Fibroblasts. Plast. Reconstr. Surg..

[62] Raica M., Cimpean A.M. (2010). Platelet-Derived Growth Factor (PDGF)/PDGF Receptors (PDGFR) Axis as Target for Antitumor and Antiangiogenic Therapy. Pharmaceuticals.

[63] Hosnuter M., Aslan C., Isik D., Caliskan G., Arslan B., Durgun M. (2017). Functional assessment of autologous platelet-rich plasma (PRP) after long-term storage at -20 °C without any preservation agent. J. Plast. Surg. Hand Surg..

[64] Moore G., Maloney J., Archer R., Brown K., Mayger K., Bromidge E., Najafi M. (2017). Platelet-rich Plasma for Tissue Regeneration Can Be Stored at Room Temperature for at Least Five Days. Br. J. Biomed. Sci..

[65] Kim J.I., Bae H.C., Park H.J., Lee M.C., Han H.S. (2020). Effect of Storage Conditions and Activation on Growth Factor Concentration in Platelet-Rich Plasma. J. Orthop. Res..

[66] Zou J., Mo X., Shi Z., Li T., Xue J., Mei G., Li X. (2016). A Prospective Study of Platelet-Rich Plasma as Biological Augmentation for Acute Achilles Tendon Rupture Repair. BioMed Res. Int..

[67] Schulz K.S., Ash K.J., Cook J.L. (2019). Clinical outcomes after common calcanean tendon rupture repair in dogs with a loop-suture tenorrhaphy technique and autogenous leukoreduced platelet-rich plasma. Vet. Surg..

